# Utility of ChatGPT as a preparation tool for the Orthopaedic In‐Training Examination

**DOI:** 10.1002/jeo2.70135

**Published:** 2025-01-02

**Authors:** Dhruv Mendiratta, Isabel Herzog, Rohan Singh, Ashok Para, Tej Joshi, Michael Vosbikian, Neil Kaushal

**Affiliations:** ^1^ Department of Orthopaedic Surgery Rutgers New Jersey Medical School Newark New Jersey USA

**Keywords:** American Academy of Orthopaedic Surgeons (AAOS), Chat Generative Pre‐Trained Transformer (ChatGPT), Orthopaedic In‐Training Exam (OITE), resident education

## Abstract

**Purpose:**

Chat Generative Pre‐Trained Transformer (ChatGPT) may have implications as a novel educational resource. There are differences in opinion on the best resource for the Orthopaedic In‐Training Exam (OITE) as information changes from year to year. This study assesses ChatGPT's performance on the OITE for use as a potential study resource for residents.

**Methods:**

Questions for the OITE data set were sourced from the American Academy of Orthopaedic Surgeons (AAOS) website. All questions from the 2022 OITE were included. All questions, including those with images, were included in the analysis. The questions were formatted in the same manner as presented on the AAOS website, with the question, narrative text and answer choices separated by a line. Each question was evaluated in a new chat session to minimize confounding variables. Answers from ChatGPT were characterized by whether they contained logical, internal or external information. Incorrect responses were further categorized into logical, informational or explicit fallacies.

**Results:**

ChatGPT yielded an overall success rate of 48.3% based on the 2022 AAOS OITE. ChatGPT demonstrated the ability to apply logic and stepwise thinking in 67.6% of the questions. ChatGPT effectively utilized internal information from the question stem in 68.1% of the questions. ChatGPT also demonstrated the ability to incorporate external information in 68.1% of the questions. The utilization of logical reasoning (*p* < 0.001), internal information (*p* = 0.004) and external information (p = 0.009) was greater among correct responses than incorrect responses. Informational fallacy was the most common shortcoming of ChatGPT's responses. There was no difference in correct responses based on whether or not an image was present (*p* = 0.320).

**Conclusions:**

ChatGPT demonstrates logical, informational and explicit fallacies which, at this time, may lead to misinformation and hinder resident education.

**Level of Evidence:**

Level V.

**Abbreviations**
AAOS: American Academy of Orthopaedic Surgeons
ChatGPT: Chat Generative Pre‐Trained Transformer
LLM: large language model
OITE: Orthopaedic In‐Training Exam
PGY: postgraduate year
PSITE: Plastic Surgery In‐Service Examination

## INTRODUCTION

Examinations at the resident level are vital in evaluating future success, especially in surgery [29]. Performance on in‐training examinations is predictive of future board certification [16, 27]. Conversely, poor performance on in‐training examinations is associated with resident burnout [26]. For the Orthopaedic In‐Training Examination (OITE), eleven domains are captured: basic science, foot and ankle, hand, hip and knee, oncology, paediatrics, shoulder and elbow, spine, sports medicine, trauma and practice management [18]. The breadth of knowledge required for the OITE emphasizes the importance of understanding not only the procedural aspect of an individual's training, but also the growing research behind it. Hence, the resources used for exam preparation are under constant scrutiny due to the constantly evolving nature of the information that the questions are based on. While reviewing past examinations had the most positive impact on OITE score [23], explanations will change from year to year, prompting residents to seek updated information from supplementary resources.

The recent introduction of Chat Generative Pre‐Trained Transformer (ChatGPT) may have implications as a novel educational resource when studying for the OITE. ChatGPT uses deep learning techniques to generate human‐like responses to user prompts. At the medical student level, ChatGPT has already been shown to achieve a passing score on USMLE Step 1 and Step 2‐CK, while providing applicable context and explanations for all questions [11]. At the resident education level, ChatGPT has been utilized to answer the Plastic Surgery In‐Service Examination (PSITE) from 2022, as well as the PSITEs from recent years [13, 15]. Given its validity as a resource in other realms of medicine, we aim to assess ChatGPT's performance on the OITE, ability to provide reasoning, and fallacies through evaluation of the generated responses.

## MATERIALS AND METHODS

The questions comprising the data set were sourced from the American Academy of Orthopaedic Surgeons (AAOS) website, which is a recognized and accepted question bank for OITE preparation. To cover the evolving orthopaedic surgery curriculum, all questions from the 2022 OITE were included. ChatGPT 3.5, developed by OpenAI, was used for this study.

All questions, even those with visual content, were included in the analysis. The questions were formatted in the same manner as presented on the AAOS website, with the question, narrative text and answer choices separated by a line. Each question was evaluated in a new chat session to minimize confounding variables, considering ChatGPT's ability to learn from mistakes and gather external information. ChatGPT has the ability to generate texts based on previous inputs [21].

The questions and their corresponding answers, along with the analysis, were copied from the AAOS OITE website into a Microsoft Word document. Once ChatGPT provided responses, they were added to a shared Microsoft Word document. Two independent reviewers evaluated each response, and any discrepancies were resolved by a third reviewer. The responses provided by ChatGPT were assessed based on the principles of natural coherence [28], which consider the prevalence of the following three characteristics in the responses:

- Logical reasoning: The system employed logical thinking and stepwise analysis to generate responses based on the information provided in the question stem.
- Internal information: The system utilized the information available within the question stem to derive appropriate responses.
- External information: The system incorporated external knowledge, including references to literature, to support its responses.

This method has been previously used in a similar study by Gupta et al. [13].

For instance, for a question regarding a post‐hip arthroplasty patient presenting with persistent limp with torso tilting toward the ipsilateral surgical side, ChatGPT selected the correct answer with the response: ‘This patient likely has abductor muscle weakness resulting in a Trendelenburg gait. This is a common complication following total hip arthroplasty and may lead to early implant failure if not addressed. The initial treatment is usually conservative with observation, non‐steroidal anti‐inflammatory drugs, and physical therapy to strengthen the abductor muscles. If conservative measures fail, operative intervention may be necessary. Magnetic resonance imaging or metal ion level testing may be indicated if an adverse local tissue reaction is suspected, but it is not the initial step in management for this patient's presentation. A tendon transfer would not be the first‐line treatment for this condition. Bone scan is not indicated for this patient's presentation’. By proceeding in a stepwise fashion from the physical exam finding in the question (internal information) to deducing that the patient's treatment will likely be conservative, ChatGPT displayed logical reasoning. Further, by assessing potential complications, it exhibited its ability to call upon external information to answer the question correctly. For an incorrect response, the following categories were used, as per a similar study [13]:

- Logical fallacy: The response demonstrated logical thinking but failed to provide an accurate answer to the question.
- Informational fallacy: The response displayed logical reasoning but missed an essential piece of information from the question stem necessary for a correct answer.
- Explicit fallacy: The response lacked logical reasoning and failed to utilize the information in the question stem to answer correctly.

For this categorization, only one option could be selected. An example of an explicit fallacy response was ‘Positive alpha‐defensin test is a major criterion for infection in the 2018 definition of periprosthetic hip and knee infection’, where ChatGPT did not use any logic or internal information and gave a randomly generated output. An instance in which logical fallacy occurs is when ChatGPT's logic makes an accurate interpretation of the problem but fails to reach proper treatment: ‘The symptoms described in the scenario are suggestive of dorsal radial wrist impingement, also known as intersection syndrome. In this condition, the extensor pollicis longus and abductor pollicis longus tendons in the second dorsal compartment become inflamed and irritated as they cross over the tendons of the extensor carpi radialis brevis and longus in the first dorsal compartment. Therefore, it is most appropriate for surgery to be directed at the release of the first dorsal wrist compartment tendon sheath’. The answer, however, was to perform a release of the second dorsal wrist compartment due to the tendons originating from the second compartment resulting in the tenosynovitis/bursitis. Specific questions could not be included due to copyright concerns. Data analysis, including accuracy assessment and statistical analysis, was performed using Microsoft Excel. To test for the precision of responses, five simulations were conducted in addition to the first simulation. To detect variation, a one‐sample *t* test was used. Unpaired chi‐square tests were conducted to evaluate the statistical significance of categorical variables, with a significance level of *p* < 0.05. Mean comparisons were made using one‐sample or independent samples *t* tests where appropriate. In order to generate percentile data by postgraduate year (PGY) level, the OITE Technical Report from 2022 was used as the benchmark (https://www.aaos.org/globalassets/education/product-pages/oite/oite-2022-technical-report-20230125.pdf).

## RESULTS

### ChatGPT performance

ChatGPT was utilized to answer a total of 207 questions in the OITE data set. Among the 207 questions, ChatGPT provided accurate responses to 100 questions, yielding an overall success rate of 48.3% based on the 2022 AAOS OITE. A manual simulation of ChatGPT was run five times, which yielded the following number of questions correct: 104 (50.2%), 103 (49.8%), 99 (47.8%), 102 (49.3%) and 99 (47.8%). This is an average of 101.4 questions correct out of 207 (49.0%). A one‐sample *t* test comparing our hypothetical mean (100 questions) to the simulation yielded no statistical significance (*p* = 0.246). Using ‘C’ as the standard answer choice yielded a 21.3% success rate, which was significantly different from our simulation results (*p* < 0.001). Using the OITE technical report, ChatGPT‐3.5 performed at a 34th percentile PGY‐1 in the test taken in 2022. However, there was a drop‐off for every year thereafter until it performed below the first percentile at the PGY‐4 and ‐5 levels. These findings are illustrated in Table 1.

**Table 1:** 2022 Performance on OITE by year.

	ChatGPT‐3.5	PGY‐1	PGY‐2	PGY‐3	PGY‐4	PGY‐5
Mean number of questions correct (out of 275 (SD))	134.75	143.55 (20.91)	159.69 (17.69)	175.87 (23.27)	185.72 (17.48)	191.55 (16.61)
Percentage correct	49	52.2	58.1	64	67.5	69.7
GPT‐3.5 percentile rank vs. cohort	‐	34	8	4	<1	<1

Abbreviations: ChatGPT, Chat Generative Pre‐Trained Transformer; OITE, Orthopaedic In‐Training Exam; PGY, postgraduate year; SD, standard deviation.

### Evaluation of coherence and information utilization

ChatGPT's responses were evaluated based on the principles of natural coherence. In terms of logical reasoning, ChatGPT demonstrated the ability to apply logic and stepwise thinking in 67.6% of the questions. Furthermore, ChatGPT utilized internal information from the question stem in 68.1% of the questions. In addition to internal information, ChatGPT incorporated external information in 68.1% of the questions.

### Performance comparison

When comparing the responses for correct answers to those for incorrect answers, several factors were assessed. In terms of logical reasoning, ChatGPT employed logical thinking in 80.0% of the questions with correct responses; however, logical reasoning was observed in only 56.1% of the incorrect questions. Furthermore, internal information utilization was observed in 77.0% of the questions with correct responses and 59.8% of the questions with incorrect responses. In terms of external information, ChatGPT utilized this resource in 76.0% of the questions with correct responses and 60.1% of the questions with incorrect responses. Statistical analysis revealed that the utilization of logical reasoning (*p* < 0.001), internal information (p = 0.004) and external information (*p* = 0.009) was greater among correct responses than incorrect responses.

Regarding the impact of clinical context, when a vignette was present, ChatGPT answered 50.7% correctly compared to 42.1%, where no vignette was given. However, this was not statistically significant (*p* = 0.057). When examining whether the presence of a vignette in an image‐based question impacted the response, ChatGPT was more likely to get the answer correct (47.4% vs. 33.3%, *p* = 0.023). On the other hand, in a non‐image‐based question, ChatGPT maintained no difference in answering the question based on context (54.1% vs. 42.9%, *p* = 0.472).

### Identification of response fallacies

During the evaluation process, three types of response fallacies were identified for incorrect answers provided by ChatGPT:

- Logical fallacy: In 18.7% of the incorrect responses, ChatGPT demonstrated stepwise thinking but failed to provide an accurate answer to the question.
- Informational fallacy: Approximately 66.4% of the incorrect responses displayed logical reasoning but failed to attribute a crucial piece of information from the question stem, resulting in an incorrect answer.
- Explicit fallacy: In 15.0% of the incorrect responses, ChatGPT failed to demonstrate logical reasoning and was unable to utilize the information provided in the question stem to answer correctly.

### Impact of visual content

Among the questions included in the study, 39.1% of them presented images in the question stem. For these image‐based questions, ChatGPT provided the correct answer 46.9% of the time. On the other hand, for questions without images, ChatGPT achieved an accuracy rate of 49.2%. However, statistical analysis indicated that the difference in performance between image‐based and non‐image‐based questions was not statistically significant, with a *p* value of 0.320.

## DISCUSSION

This study demonstrates ChatGPT's efficacy in answering almost half of the questions on a 2022 AAOS OITE examination. Further, ChatGPT displayed the potential to provide logical, internal and external information on nearly two thirds of the questions it answered. When it answered incorrectly, it was most frequently due to an informational error, as opposed to a logical or explicit error. Gupta et al. produced similar findings in a study conducted on the 2022 PSITE wherein the total accuracy of answers was 54.96%, and external information was used more frequently in correct answers [13]. By delineating the shortcomings of ChatGPT's responses, residents may be better advised on how to best utilize this tool in their OITE preparation.

In a recent study, Lum et al. posited that ChatGPT performed at the level of a 40th percentile PGY‐1 resident on the OITE, but fell below the 10th percentile when compared to all subsequent years [19]. Our study corroborated these findings. Given the structure of an orthopaedic residency intern year, wherein residents are required to rotate in non‐orthopaedic specialities, a significant lack of exposure to surgical techniques may result in a shortage of information, contributing to the comparable performance of ChatGPT [8].

ChatGPT demonstrated logical reasoning in 67.6% of all questions and included internal information from the prompt in 68.1% of all questions, incorporating both factors significantly more often when providing correct answers. These factors highlight ChatGPT's ability to comprehend questions and provide individualized feedback; the greater prevalence of correct answers indicates that stepwise rationale and identification of key details strongly influence the accuracy of the model [1, 9]. When considering the inclusion of external information, ChatGPT provided sources from the literature in approximately 68% of all explanations, notably more significantly in correct answers. Increased familiarity with the literature has been associated with improved resident OITE performance; thus, the incorporation of external sources contributing to increased accuracy of the model is comprehensible [10, 30]. However, given ChatGPT's known shortcomings in fabricating literature, it is difficult to assess the true source of the information [4, 12, 24]. Future studies should compare the frequency of true sources and fictional sources to help elicit the model's tendency to fabricate when providing answers and explanations to resident‐level questions.

Interestingly, ChatGPT did not demonstrate increased difficulty in answering image‐based questions, a result which has been corroborated by its performance on the Radiology Board Examination [5]. Given a clinical vignette with an image, ChatGPT was more likely to get the answer correct as opposed to when no context was given. Other studies have omitted image‐based questions when utilizing ChatGPT‐3.5, as it is difficult to draw conclusions regarding whether the accuracy of the model is due to efficacy or hallucination [3]. The use of ChatGPT‐4 has been acknowledged as a solution to this limitation as the updated platform provides the ability to input images when providing prompts, improving the accuracy of the model in answering image‐based questions [2].

In addition to characterizing correct responses provided by ChatGPT to understand factors influencing the model's accuracy, it is equally important to understand the limitations of the model by exploring the incorrect answers provided. While only approximately one‐fifth of the incorrect responses encountered a logical error, the fallacy indicates a major gap in the model. In these incorrect responses, ChatGPT provided a correct methodology for answering the question, however, was unable to elicit a correct response at the final step. The prevalence of logical fallacy highlights ChatGPT's established shortcoming of providing logical reasoning yet reaching inaccurate conclusions leading to misinformation [7, 17]. Moreover, the majority of incorrect responses demonstrated informational fallacy, in which the model supplied stepwise reasoning but failed to incorporate key details from the prompt to provide a correct answer. ChatGPT's inability to correctly identify pertinent information while maintaining stepwise thinking has also been demonstrated on a medical‐student level parasitology examination and a board‐level ophthalmology examination, indicating a key shortcoming of the model in determining relevant information especially in a medical context [14, 22]. In less than one‐fifth of the incorrect answers, ChatGPT demonstrated explicit fallacies where the model failed to provide either logical reasoning or key details from the question, emphasizing ChatGPT's tendency to hallucinate. A major concern with ChatGPT is its ability to provide fabricated information that seems to be reputable, particularly pertaining to science [6, 20, 25]. The occurrence of explicit fallacies, albeit the frequency, increases vulnerability to misinformation which may potentially harm resident education. The recent development of ChatGPT‐4 provides a possible improvement on the current limitations of ChatGPT‐3.5.

As it currently stands, however, ChatGPT cannot serve as a standalone tool in the preparation for OITE examinations. Currently, a concern amongst orthopaedic educators is the rudimentary or superficial understanding of concepts in orthopaedic surgery, due to shift away from textbooks and research articles toward condensed guides and flashcards. Hence, the utilization of ChatGPT as another flashcard‐like modality would feed into the current memorization paradigm that exists in medical education. However, a potential utility of this resource would be to further explain correct or incorrect answers provided with the proper prompting information, which would incorporate evidence‐based articles to further learning. Due to the ever‐changing field of orthopaedic surgery and rapidly evolving techniques and guidelines, ChatGPT has the potential to serve as a foundational resource than current question banks, due to their growing obsolescence by day.

Limitations of this study include the use of only one OITE practice exam. Had this study been reproduced with multiple practice sets, it may have held more external validity. Further, bias on the part of the researcher may have influenced these findings, as ChatGPT could have used input from previous questions to formulate an answer for later questions. To mitigate this, a new conversation was utilized for each simulation that was performed. However, it is still uncertain whether ChatGPT has access to the other conversations. Overall, this allowed for improved internal validity. Finally, the omission of some elements of the question (tables or images) may yield erroneous results. Future studies should reproduce this study with newer large language models (LLMs) to mitigate these obstacles.

## CONCLUSION

Preparation for the OITE is important for the success of orthopaedic surgery residents. LLMs, such as ChatGPT‐3.5, provide a unique learning resource that can improve resident education. However, ChatGPT currently represents a poor source of information reliability for medical education and cannot be recommended as a study source.

## AUTHOR CONTRIBUTIONS

Dhruv Mendiratta, Isabel Herzog and Rohan Singh were responsible for the research design, acquisition, analysis and interpretation of data. Dhruv Mendiratta, Isabel Herzog, Rohan Singh, Ashok Para, Tej Joshi, Neil Kaushal and Michael Vosbikian were responsible for drafting and critical revision of the manuscript. All authors have read and approved the final submitted manuscript.

## CONFLICT OF INTEREST STATEMENT

The authors declare no conflicts of interest.

## ETHICS STATEMENT

This study did not require ethical approval, as per institutional guidelines. Since there were no patients in this study, no informed consent was required.

## Data Availability

Data are available upon reasonable request to the authors.
